# Radiotherapy alone for stage IE ocular adnexal mucosa-associated lymphoid tissue lymphomas: long-term results

**DOI:** 10.1186/s13014-020-1477-8

**Published:** 2020-01-30

**Authors:** Masanari Niwa, Satoshi Ishikura, Kotoha Tatekawa, Natsuko Takama, Akifumi Miyakawa, Toshinobu Kubota, Eriko Kato, Yuta Shibamoto

**Affiliations:** 1 Department of Radiology, Nagoya City University Graduate School of Medical Sciences, 1 Kawasumi, Mizuho-cho, Mizuho-ku, Nagoya, 467-8601 Japan; 2 Department of Radiology, Ikeda City Hospital, 3-1-18 Jyonan, Ikeda, Osaka, 563-8510 Japan; 3 Department of Radiation Oncology, National Hospital Organization Nagoya Medical Center, 4-1-1 Sannomaru, Naka-ku, Nagoya, 460-0001 Japan; 4 Department of Ophthalmology, National Hospital Organization Nagoya Medical Center, 4-1-1 Sannomaru, Naka-ku, Nagoya, 460-0001 Japan

**Keywords:** Ocular adnexal, MALT lymphoma, Radiation therapy, Cataract

## Abstract

**Background:**

To evaluate the long-term efficacy and toxicity of radiation therapy in patients with Stage IE primary ocular adnexal mucosa-associated lymphoid tissue lymphoma.

**Methods:**

We designed a retrospective analysis to evaluate 81 patients with ocular adnexal mucosa-associated lymphoid tissue lymphoma treated with radiation therapy between 2006 and 2016. The median radiation dose was 30 Gy (range, 30–36 Gy in 15–18 fractions). Local control, progression-free survival, overall survival, and cumulative incidence of Grade 3 cataract were calculated by using the Kaplan–Meier method.

**Result:**

The median follow-up time was 74 months (range, 4–157 months). The 5-year local control was 100%. Although local relapse was suspected in 3 patients after radiation therapy, 2 patients were pathologically diagnosed as IgG4-related inflammation and in 1 patient as intense inflammatory cell infiltration. The 5-year progression-free survival was 94.4%. Five patients had relapse at distant sites. The 5-year overall survival was 98.8%. Twenty patients had Grade 3 cataract. The 5-year cumulative incidences of Grade ≥ 3 and Grade ≥ 2 cataract for 58 patients treated without a lens shield were 38 and 40%, respectively. The incidence of Grade ≥ 3 cataract was 42% for 50 patients treated with 6-MV X-rays (estimated lens dose: 29 Gy) and 17% for 8 patients treated with 9-MeV electrons (estimated lens dose: 24 Gy).

**Conclusions:**

Radiation therapy alone yielded excellent local control and long-term survival in Stage IE ocular adnexal mucosa-associated lymphoid tissue lymphoma. Long-term observation with careful attention to relapse at distant sites is necessary. In the case of suspected local relapse, IgG4-related disease should be carefully ruled out.

## Background

The concept of mucosa-associated lymphoid tissue (MALT) lymphoma was originally described by Isaacson and Wright [1] in 1983, but it was not recognized at that time. Thereafter, the distinct clinical-pathological and molecular features of MALT lymphoma were gradually accepted. MALT lymphoma is now classified as extranodal marginal zone B-cell lymphoma in the World Health Organization (WHO) classification [2, 3]. MALT lymphoma is the commonest marginal zone B-cell lymphoma and accounts for 5–8% of all B-cell lymphomas [4, 5]. MALT lymphoma occurs in the ocular adnexa, salivary gland, thyroid gland, lung, breast, stomach, and colon. The most common site of MALT lymphoma is the stomach [6, 7]. Several factors associated with MALT lymphoma have been identified including infectious microorganisms, particularly *Helicobacter pylori*, related to gastric MALT lymphoma [8, 9]. Recently, *Chlamydia psittaci* infection has been identified as a pathogenesis of ocular adnexal MALT lymphoma [10]. *Chlamydia psittaci* not only induces lymphoid proliferation, but also seems to be responsible for chromosomal aberration, probably due to its mitogenic activity or indirectly induced oxidative damage. The incidence of ocular adnexal MALT lymphoma is rapidly increasing, with annual rates ≥6%, with no evidence of peaking [11].

Several treatment modalities exist for MALT lymphoma. In general, MALT lymphoma is a radiosensitive disease [12, 13]. For non-gastric MALT, radiation therapy (RT) is considered when symptomatic [14–17]. Other therapeutic options for ocular adnexal MALT lymphoma include systemic chemotherapy, surgery alone, and “watch and wait” strategy, but none are standard [18]. Recently, the efficacy of antibiotic therapy has been reported in localized ocular adnexal MALT lymphoma associated with *Chlamydia psittaci* infection [10]. Although RT alone for this disease achieves excellent tumor control, cataract is the most common and serious late morbidity [19]. RT alone can result in long-term complications such as dry eye syndrome and subsequent keratitis, and they cause a serious decline in quality of life [20]. Since there are only a few well-documented reports of the efficacy and morbidity of RT in this disease, we report herein our experience with ocular adnexal MALT lymphomas that were treated with involved-field RT with long-term results.

## Methods

### Patients

This was a retrospective, observational case study approved by the institutional review board. We reviewed the charts of 81 patients with a biopsy-proven diagnosis of ocular adnexal MALT lymphoma between 2006 and 2016 at a single institution. Patients with transformed lymphoma component at diagnosis were excluded. All patients had Stage IE disease. Staging assessments included blood test, positron emission tomography–computed tomography (PET-CT), bone marrow aspiration, and gastroscopy. There were 42 men and 39 women, and their median age at the start of RT was 66 years (range, 29–90 years). Among the 81 patients, 57 had a tumor located in the orbit, 21 had a tumor in the conjunctiva, and 3 had a lacrimal gland tumor. Bilateral orbital involvement was observed in 9 patients.

### Radiotherapy

All patients were treated with RT alone. The clinical target volume (CTV) of all patients included the entire orbit of the affected side. The planning target volume (PTV) included the CTV with a 5-mm margin. The median radiation dose was 30 Gy (range, 30–36 Gy in 15–18 fractions).

Six-MV photon beams with a wedged pair of fields were used for 50 patients. Nine-MeV electron beams with a single anterior field were used for 26 patients, and a 16-MeV electron beam was used for 1 patient. Combinations of 6-MV photon and 9-MeV electron beams were used for 4 patients. Nineteen patients were treated with a lead lens block. Nine patients with bilateral lesions were treated simultaneously.

### Follow-up evaluation and statistical analysis

At follow-up visits, physical examinations and blood tests were performed. PET-CT was performed at least once every 5 years. RT-related adverse events were graded according to the National Cancer Institute-Common Terminology Criteria for Adverse Events (NCI-CTCAE, version 4.0). Cataract Grade 2 was defined as symptomatic with moderate decrease in visual acuity, and Grade 3 was defined as symptomatic with marked decrease in visual acuity or operative intervention indicated. The endpoints of our study were local control (LC), progression-free survival (PFS), overall survival (OS), and incidence of adverse events calculated from the date of starting RT to the event. The rates were calculated using the Kaplan–Meier method. Differences in the incidence of cataract between the X-ray and electron beam groups were examined using the log-rank test. Local relapse was considered as an event for LC. First progression and death from any cause were considered as an event for PFS, and death from any cause for OS. Complications were graded according to the Common Terminology Criteria for Adverse Events Version 4. All statistical analyses were performed using EZR (Saitama Medical Center, Jichi Medical University, Saitama, Japan), which is a graphical user interface for R (The R Foundation for Statistical Computing, Vienna, Austria). More precisely, it is a modified version of R commander designed to add statistical functions frequently used in biostatistics [21].

## Results

**Fig. 1:**
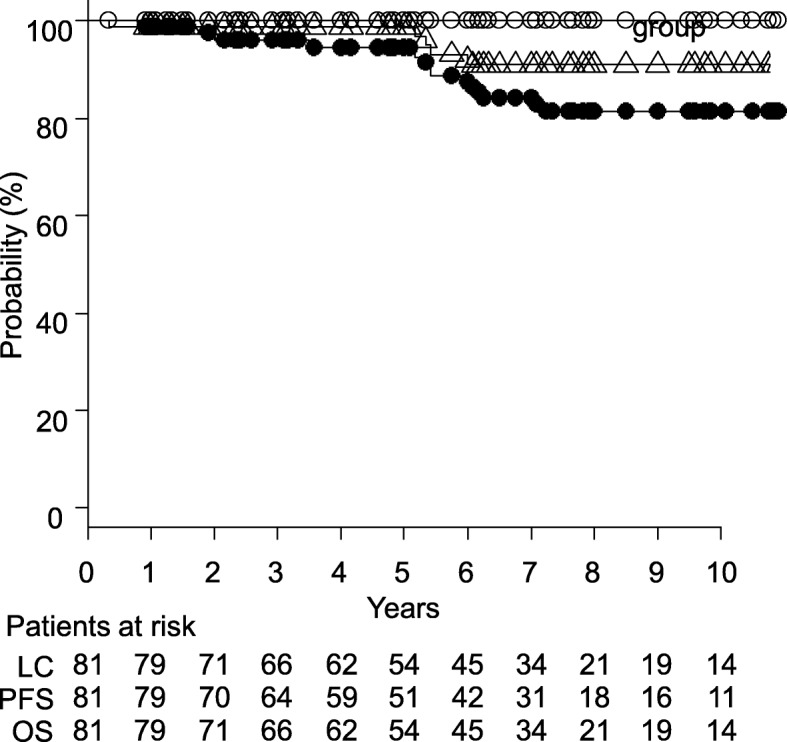
Curves of local control (LC, open circle), progression-free survival (PFS, filled circle), and overall survival (OS, triangle) for 81 patients with orbital MALT lymphoma

Patient characteristics are shown in Table 1. Two patients had surgery before RT. One underwent an excision of tumor and the other underwent a conjunctival resection. No patients received cytotoxic chemotherapy or antibiotics before or during RT. The median follow-up period was 74 months (range, 4–157 months). Fifty-four patients had a complete follow-up of 5 years. Of the 81 patients, 56 had a complete response, 15 had a partial response, and 3 had a stable disease. In 7 patients, their responses could not be classified, but none had a progressive disease. The plots of LC, PFS, and OS are shown in Fig. 1. The 5-year LC was 100% (Fig. 1). Although local relapse was suspected in 3 patients after RT, 2 patients were pathologically diagnosed with IgG4-related inflammation and 1 patient with intense inflammatory cell infiltration. One of the 2 patients diagnosed with IgG4-related inflammation was observed without treatment and has shown no progression. The other had nodules in the right parotid gland and right lung 4 years after being diagnosed with IgG4-related inflammation, which were pathologically diagnosed as diffuse large B-cell lymphoma (DLBCL); the patient maintains complete response with chemotherapy consisting of rituximab, cyclophosphamide, doxorubicin hydrochloride, vincristine, and prednisolone (R-CHOP). The patient diagnosed with intense inflammatory cell infiltration has shown no progression.

**Table 1:** Patient characteristics

Characteristic	No. of patients
Sex	
Male	42
Female	39
Median age (range) at the start of RT, years	66 (29–90)
Bilateral presentation	9
Primary site	
Orbit	57
Conjunctiva	21
Lacrimal gland	3

The 5-year PFS was 94.4% (Fig. 1). Table 2 summarizes the clinical courses of 5 patients who had relapse at distant sites. The median time to relapse was 42 months. The relapses occurred at various sites including the contralateral conjunctiva, parotid gland, cervix, lung, intraperitoneal cavity, and femur. Of these 5 patients, 3 were treated with chemotherapy. Two of the 3 patients were treated with R-CHOP and have maintained complete response. The other patient was treated with one course of R-CHOP but chemotherapy was then switched to rituximab alone due to hemorrhagic cystitis and ileus. She subsequently had a relapse in the mediastinum and was treated with rituximab and 5-fluorouracil, and she has maintained complete response for 30 months. The other 2 patients who did not receive chemotherapy have been observed without treatment and shown no further progression.

**Table 2:** Details of 5 patients with distant relapse

Age (years)/ sex	Primary tumor site	Recurrence at distant sites	Years to recurrence/ follow-up (years)/status	Treatment/local status
71/M	Orbit	Contralateral conjunctiva	6.2/6.3/alive	Observation/no change
69/F	Orbit	Fossa axillaris, hilum of lung, mediastinal lymph node, peritoneum	2/10.3/alive	R-CHOP → rituximab alone/CR → relapse (8 years later)
52/F	Conjunctiva	Contralateral conjunctiva	7.1/12.3/alive	Observation/no change
67/M	Conjunctiva	Intestinal membrane, skin	1.8/7.7/alive	R-CHOP/CR
63/M	Conjunctiva	Left hilus of kidney	3.7/10.3/alive	R-CHOP/CR

*Abbreviations: M* male, *F* female, *CR* complete response; R-CHOP = rituximab, cyclophosphamide, doxorubicin hydrochloride, vincristine, and prednisolone

The 5-year OS was 98.8% (Fig. 1). There were five deaths. Three of the five deaths were due to unrelated causes (gastric cancer, exacerbation of interstitial pneumonia, and choking on food, respectively). Of the remaining two, one was found to have died in the prognostic survey and the other was found dead at home; reasons for the deaths in these patients were unknown. There were no deaths due to MALT lymphoma relapse or treatment complications.

**Fig. 2:**
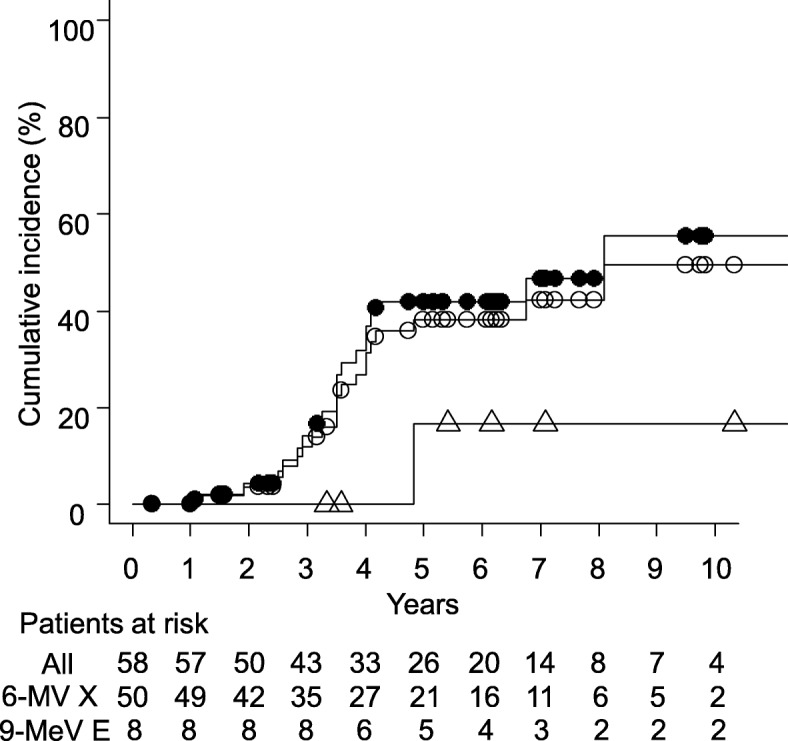
Cumulative incidence of Grade ≥ 3 cataract in all 58 patients treated without a lens shield (open circle), in 50 patients treated with 6-MV X-rays (filled circle), and in 8 patients 9-MeV electrons (triangle). The median age at the start of RT was 69 (range, 90–29), 70 (range, 90–43), and 64 (range, 88–29) years, respectively

One patient incidentally had retinal detachment during RT and then underwent surgery. A lens block used was an external eye shield. The tumor was in the conjunctiva around the cornea and not massive. It was apart from the retina. Thus, we concluded that the retinal detachment was not related to the RT technique or tumor shrinkage. There were no other Grade ≥ 3 acute morbidities. Nine patients had Grade 2 late morbidity (dry eye, 3; superficial punctate keratitis, 4; cataract, 2). Twenty patients had Grade 3 cataract. Otherwise there were no Grade ≥ 3 late adverse events. We excluded patients diagnosed with age-related cataracts and patients diagnosed with bilateral cataracts at the same time after RT to one side. The 5-year cumulative incidences of Grade ≥ 3 and Grade ≥ 2 cataract for 58 patients treated with 6-MV X-rays or 9 MeV electrons without a lens shield were 38% (95% confidence interval [CI], 22–51%) and 40% (95% CI, 24–53%), respectively (Fig. 2). The incidence of Grade ≥ 3 cataract was 42% (95% CI, 24–55%) for 50 patients treated with 6-MV X-rays (prescribed dose: 30–31 Gy) and 17% (95% CI, 0–42%) for 8 patients treated with 9-MeV electrons (prescribed dose: 30 Gy); the difference was not significant (*P* = 0.59). The median age at the start of RT for patients with Grade 3 cataract was 67 years (range, 50–80 years). Of the 9 patients who received RT on both eyes, 5 were treated with 6-MV X-rays or 9-MeV electrons without a lens shield. Of them, one patient who had received RT at 76 years old underwent bilateral cataract surgery at the same time (73 months after RT), but was diagnosed with age-related cataract. One patient who had received RT at 67 years old had Grade 3 cataract on left side 30 months after RT, and another patient who had received RT at 61 years old had Grade 2 cataract on right side 69 months after RT. They did not develop cataract on the other side.

## Discussion

In general, patients with MALT lymphoma have a better prognosis than those with other eye lymphomas. Olsen et al. [22] reported on 797 patients with a histologically verified orbital lymphoma and showed that extranodal marginal zone B-cell lymphoma and follicular lymphoma were associated with a markedly better prognosis (10-year disease-specific survival of 92 and 71%, respectively) than DLBCL and mantle cell lymphoma (10-year disease-specific survival of 41 and 32%, respectively). Our results showed that ocular adnexal MALT lymphoma responded quite well to moderate-dose RT, with good LC and long-term survival. Similar results were shown in previous reports. Joo et al. [23] treated 134 MALT lymphoma patients using definitive RT. Among the 88 orbital MALT lymphoma patients, the median dose was 30.6 Gy. The 5-year LC and relapse-free survival (RFS) rates were 94 and 80%, respectively. Harada et al. [24] treated 86 patients with histologically proven Stage I primary ocular adnexal MALT lymphoma. The median dose was 30 Gy. The 5- and 10-year OS rates were 97.6 and 93.5%, and the 5- and 10-year local RFS were 97.0 and 90.8%, respectively. Ohga et al. [25] treated 73 patients with Ann Arbor stage IE ocular adnexal MALT lymphoma. The median dose was 30 Gy. The 5-years LC and PFS were 100 and 81.5%, respectively.

In our study, no local relapse was observed. Although local relapse was suspected in 3 patients after RT, 2 patients were diagnosed by biopsy with IgG4-related inflammation and 1 patient with intense inflammatory cell infiltration. One of the 2 patients diagnosed with IgG4-related inflammation developed DLBCL of the right parotid gland and right lung 4 years later. We did not include this patient in the events for local or distant relapse of MALT lymphoma, but there might have been DLBCL with a background of MALT lymphoma at the time of diagnosis or it might have been large cell transformation of MALT lymphoma. There have been some reports of large cell transformation in gastric MALT lymphoma, but such transformation in ocular adnexal MALT lymphoma is rare [26]. Because MALT lymphoma and IgG4-related diseases are immunohistochemically similar [27], we should be careful when local relapse is suspected.

Although the LC rate has been reported to be over 95%, distant relapse occurs in 8–23% and local RT might not be useful in preventing distant relapse [12, 13, 28, 29]. Tsang et al. [13] showed that RT alone yielded excellent LC with infrequent long-term toxicity, but the risk of distant relapse was a problem. For the management of MALT lymphoma, systemic disease control with initial chemotherapy in addition to LC may be controversial. The benefit of combining RT and chemotherapy has not been investigated well in localized MALT lymphoma. Hashimoto et al. [30] showed that the combination of RT and rituximab tended to lead to better RFS than RT alone. However, MALT lymphoma has a good prognosis after initial treatment with RT alone, and we think that upfront chemotherapy is not mandatory and can be considered as salvage treatment at relapse. If relapse is limited to a few lesions, RT may be considered as an option alternative to chemotherapy. Favorite sites of relapse were reported to be contralateral paired organs and distant mucosal sites rather than the primary tumor site [23, 31, 32]. We also observed relapses in the contralateral conjunctiva, fossa axillaris, hilum of lung, mediastinal lymph node, peritoneum, intestinal membrane, skin, and left hilus of kidney. There were 2 patients who relapsed in the contralateral conjunctiva. In the 3 patients diagnosed with distant relapse and treated with chemotherapy, 2 patients treated with R-CHOP achieved complete response, but the other patient treated with rituximab alone had relapse in the mediastinum. Our results showed that R-CHOP was effective for distant relapse. Long-term observation with careful attention to relapse at distant sites is necessary.

Regarding adverse events, the incidences of Grade ≥ 2 and ≥ 3 cataract were 40 and 38%, respectively. Previous studies showed that more than half of patients receiving RT without a lens shield had cataract within 3–9 years following RT. [ 30, 33] Cataract is the most common late morbidity in ocular adnexal MALT lymphoma patients treated with RT. The risk of cataract and the latent period between RT and appearance of cataract are dose dependent. Emami et al. [34] estimated TD 5/5 (probability of 5% complication within 5 years from RT) of the lens for Grade ≥ 3 cataract to be 10 Gy and TD 50/5 (probability of 50% complication within 5 years from RT) to be 18 Gy. In our study, the lens of most patients treated with 6-MV photon beams received about 95% of the prescribed dose, i.e., 29 Gy with a 1.9 Gy daily dose, and those treated with 9-MeV electron beams received about 80% of the prescribed dose, i.e., 24 Gy with a 1.6 Gy daily dose. Thus, the observed incidence of Grade ≥ 3 cataract was lower than expected. To our knowledge, the cumulative incidences of cataracts for X-rays and electrons with estimated absorbed doses in the lens have not been reported. Accumulation of such data may lead to revision of the probability of complications from RT. The median time between RT and the appearance of Grade 3 cataract was 3.5 years (range, 1.1–8.1 years) and thus longer follow-up of more than 5 years is needed. Park et al. [19] showed that consideration of RT-related factors such as lens protection and RT dose may reduce the risk of radiation-induced cataract. In cataract surgery, the cloudy lens is removed and an intraocular lens is inserted. Invasiveness of the surgery is mild, and facilities that can perform the operation as day surgery are increasing. When the lesion is close to the lens, we can irradiate the target with the appropriate dose rather than using a lead lens block to protect the lens.

Treatment options for ocular adnexal MALT lymphoma include systemic therapies such as chemotherapy and antibiotics in addition to local therapies such as RT and surgery. Jeon et al. [18] reported treatment outcomes of 208 patients with ocular MALT lymphoma treated by various modalities. With a median follow-up period of 70 months, PFS was reported to be 69.7% at 13 years, and there were no differences in survival outcomes between patients treated with RT and those treated with rituximab-containing chemotherapy (PFS, about 80% at 10 years for both groups), although the latter group had more advanced stages of this disease. They suggested that chemotherapy is recommended for younger patients, and RT is recommended for older and chemotherapy-ineligible patients from the view point of late adverse events. Ferreri AJ et al. [35] treated 27 patients with ocular adnexal MALT lymphoma with oral doxycycline. Lymphoma regression was observed in 64% of *Chlamydia psittaci* DNA–positive patients (seven of 11 experienced regression) and 38% of *Chlamydia psittaci* DNA–negative patients (6 of 16 experienced regression). In addition to RT and chemotherapy, antibiotics may be used in most ocular adnexal MALT lymphoma patients, independently of the diagnosis of *Chlamydia psittaci* infection [10]. Thus, our results compare favorably with those of other treatment modalities.

Generally, the total dose for conventional RT for MALT lymphoma is 30 Gy [12, 30]. To determine a lower effective dose may be a future task in the treatment of ocular adnexal MALT lymphoma. In recent studies, excellent LC was obtained with reduced doses [36]. Tran et al. [37] reported that the 2- and 5-year LC rates were 100 and 92%, respectively, using RT with 24–25 Gy. Timothy et al. [38] recommended delivery of 20–30 Gy at 1.5–2.0 Gy per fraction for low-grade lymphoma. The NCCN guidelines suggested that involved site RT (24–30 Gy) is recommended for stage I–II non-gastric MALT lymphomas, and lower doses for orbit involvement [39]. Recently, Pinnix et al. used ultra-low-dose (4 Gy with a 2-Gy daily dose) and 19 of 22 patients had a complete response [40]. We will consider dose reduction for this disease in the future.

This study has several limitations inherent to a retrospective design, including possible patient selection due to some variations in treatment before RT, underestimation of toxicities, variations in follow-up intervals, and lack of baseline status of cataract. Due to these limitations, our results should be interpreted with caution.

## Conclusions

RT alone achieved excellent LC and long-term survival in ocular adnexal MALT lymphoma. Long-term observation with careful attention to relapse at distant sites and radiation-induced cataract is necessary. In the case that local relapse is suspected, IgG4-related disease should be carefully ruled out.

## Data Availability

The datasets used and analysed during the current study are available from the corresponding author on reasonable request.

## Abbreviations

CTV: Clinical target volume
DLBCL: Diffuse large B-cell lymphoma
LC: Local control
MALT: Mucosa-associated lymphoid tissue
OS: Overall survival
PET-CT: Positron emission tomography–computed tomography
PFS: Progression-free survival
PTV: Planning target volume
R-CHOP: Rituximab, cyclophosphamide, doxorubicin hydrochloride, vincristine, and prednisolone
RT: Radiation therapy
WHO: World Health Organization
